# Neurodegeneration and Cancer: Where the Disorder Prevails

**DOI:** 10.1038/srep15390

**Published:** 2015-10-23

**Authors:** Petr Klus, Davide Cirillo, Teresa Botta Orfila, Gian Gaetano Tartaglia

**Affiliations:** 1 Centre for Genomic Regulation (CRG), The Barcelona Institute of Science and Technology, Dr Aiguader 88, 08003 Barcelona, Spain; 2 Universitat Pompeu Fabra (UPF), 08003 Barcelona, Spain; 3 Institució Catalana de Recerca i Estudis Avançats (ICREA), 23 Passeig Lluís Companys, 08010 Barcelona, Spain

## Abstract

It has been reported that genes up-regulated in cancer are often down-regulated in neurodegenerative disorders and *vice versa*. The fact that apparently unrelated diseases share functional pathways suggests a link between their etiopathogenesis and the properties of molecules involved. Are there specific features that explain the exclusive association of proteins with either cancer or neurodegeneration? We performed a large-scale analysis of physico-chemical properties to understand what characteristics differentiate classes of diseases. We found that structural disorder significantly distinguishes proteins up-regulated in neurodegenerative diseases from those linked to cancer. We also observed high correlation between structural disorder and age of onset in Frontotemporal Dementia, Parkinson’s and Alzheimer’s diseases, which strongly supports the role of protein unfolding in neurodegenerative processes.

It has been reported that tumor suppressor p53 has physico-chemical features that are typical of prionoid proteins associated with neurodegenerative diseases^1^. This finding is particularly interesting because it suggests that common molecular properties can be linked to relatively distant diseases. As a matter of fact, a recent study by Ibáñez *et al.*^2^ shows that transcripts up-regulated in cancer are down-regulated in central nervous system (CNS) diseases and *vice versa*. In line with this finding, a risk reduction for some cancer types has been observed in patients affected by Parkinson’s^3^ and Alzheimer’s diseases^4^.

Are there common physico-chemical determinants behind comorbidities? We re-analysed the data published by Ibáñez *et al.*^2^ to understand if differential regulation of genes can be associated with specific protein features. While the original study by Ibáñez *et al.*^2^ focused on transcripts that are exclusively up-regulated in cancer and down-regulated in CNS diseases and *vice versa*^2^, our analysis deals with genes that are exclusively associated with either CNS diseases or cancer (Fig. 1). In agreement with recent experimental findings^5^ and theoretical analyses^6^,^7^,^8^,^9^, we investigated the physico-chemical properties of gene products assuming a proportionality between transcript and protein abundances.

## Results

In this work, we used the *clever*Machine approach (available at http://www.tartaglialab.com/cs_multi/submission)^10^ to analyse physico-chemical features of proteins associated with Schizophrenia, Alzheimer’s and Parkinson’s diseases as well as colorectal, lung and prostate cancers. Analysis carried out with the *boxplotter* algorithm (accessible at http://www.tartaglialab.com/boxplotter/submit; Table S1) reveals that genes up-regulated in CNS disorders code for proteins that are poorly abundant at physiological conditions (human reference proteome)^11^,^12^, indicating that expression is significantly increased in the disease state (down-regulated genes follow the opposite trend; Fig. 2A–C). By contrast, genes up-regulated in colorectal, lung and prostate cancers are associated with proteins that are already abundant in the reference proteome (down-regulated genes follow the opposite trend; Fig. 2D–F). The finding that genes associated with different diseases are constitutively expressed at specific levels suggests a link with physico-chemical features of their product products^8^,^13^. As a matter of fact, previous reports indicate that protein abundance is intrinsically constrained by solubility^8^,^9^,^14^,^15^, unfolded polypeptides are poorly expressed^16^,^17^ and nucleic-acid binding proteins are highly abundant^18^,^19^ (Table S1).

We found that structural disorder strongly differentiates cancer types and CNS diseases (p-values <10^−5^; http://www.tartaglialab.com/cs_multi/confirm/524/36563b35ee/). Evidence for this conclusion is presented in Fig. 3, where we compared 18000 genes (~75000 protein isoforms) using ten disorder predictors^10^. For each CNS disease, we found that up-regulated genes are significantly enriched in intrinsically unfolded proteins (17 out of 18 of protein sets follow the trend giving an overall signal strength of 17/18 = 0.94; p-values <10^−5^; Fisher’s exact test; Fig. 3A), while down-regulated genes contain more structured polypeptides (signal strength = 18/18), in agreement with DisEMBL disorder predictions^20^ (see *Material and Methods*). Comparing genes up- and down-regulated in cancer types and CNS diseases, we observed that structural disorder propensity anti-correlates with order-promoting features such as alpha-helix (31 out of 36 predictors show opposite trends resulting in a score of −31/36 = −0.86) and beta-sheet (−0.91) propensities. Increase in disorder is also significantly associated with depletions in burial (predictors agreement = −0.77), hydrophobicity (−0.55) and membrane propensities (−0.47)^21^. By contrast, proteins up-regulated in colorectal and lung cancer are enriched in nucleic-acid binding ability (8 out of 12 sets follow the trend, while the remaining 4/12 do not show significant enrichments; Fig. 3B), which is in line with evidence showing that transcription factors such as p53 play a major role in oncogenesis^22^. Interestingly, prostate cancer shows significant up-regulation of membrane proteins (e.g. NGEP-L), as previously reported in other studies (3 of 6 sets follow the same trend, while the remaining 3/6 do not show significant enrichments; Fig. 3C)^23^.

Gene Ontology (GO) analysis of up-regulated genes indicates that proteins containing disordered regions are associated with increased aggregation (Alzheimer’s disease: “identical protein binding”, p-value = 10^−5^) and misfolding propensities (Parkinson’s disease: “activation of signaling protein activity involved in unfolded protein response”, p-value = 10^−4^; Fig. 4 Schizophrenia: “response to unfolded protein”, p-value=10^−3^). Interestingly, a group of disordered proteins with DNA-/RNA-binding ability is up-regulated in colorectal (“RNA processing” p-value = 10^−9^), lung (“DNA repair” p-value = 10^−4^) and prostate cancers (“ribonucleoprotein complex” p-value = 10^−5^). In addition, disordered proteins are found in pathways involving p53 (e.g. colorectal cancer: “DNA damage response, signal transduction by TP53 class mediator resulting in cell cycle arrest”, p-value = 10^−5^).

GO annotations suggest that proteins containing disordered regions are abundant in colorectal, lung and prostate cancers, although their enrichment is less significant than in Schizophrenia, Alzheimer’s and Parkinson’s diseases. To test this hypothesis, we generated random groups of human genes (same number of proteins as in the original sets) and compared their features with those of cancers and CNS diseases. We found that structural disorder is indeed enriched in both up-regulated and down-regulated cancer proteins (19 out of 36 down- and up-regulated sets follow the trend and 13/16 do not show significant enrichments; p-values < 10^−5^; Figure S1A), although the signal is stronger for Schizophrenia, Alzheimer’s and Parkinson’s diseases (18/18 up-regulated gene sets are enriched in disorder and 16/18 down-regulated sets are depleted; Figure S1B), in agreement with our original findings (Fig. 3A). We also observed that nucleic acid propensities are enriched in cancers (15/18 sets show significant increase and three are non-significantly enriched) and CNS diseases (15/18 sets have significant increase and one is non-significantly enriched), but signal strength is higher for cancers (Fig. 3B).

To further investigate the intimate connection between CNS diseases and structural disorder, we analysed 428 mutations of proteins involved in Frontotemporal Dementia, Alzheimer’s and Parkinson’s diseases (available at http://www.molgen.ua.ac.be/ADMutations/ and http://www.molgen.vib-ua.be/PDMutDB/). We observed a strong correlation (Pearson’s correlation = −0.9; p-value < 10^−3^) between age of onset and disorder^24^, which, in agreement with GO analysis, indicates that reduction in folding efficiency is a key factor in neurodegeneration (Fig. 5). In line with this observation, previous reports indicate that intrinsically unfolded proteins such as α-synuclein (Parkinson’s disease^25^), Aβ_42_ (Alzheimer’s disease^26^) and DISC1 (Schizophrenia^27^) cause neuronal damages by assembling into amyloid fibrils. As proteomic analyses indicate that amyloid-forming proteins have an intrinsic propensity to attract disordered proteins^26^, it is possible that neurotoxicity arises from direct co-aggregation of proteins that have unfolded regions available for promiscuous interactions. Thus, up-regulation of disordered proteins might be the consequence of a cellular response to compensate progressive sequestration in amyloid deposits. To investigate this hypothesis, we compared proteins sequestered by amyloid fibrils^26^ and those deregulated in Alzheimer’s disease. The *clever*Machine analysis^10^ indicates that proteins binding to amyloid aggregates are not physico-chemically dissimilar to those up-regulated in Alzheimer’s disease (see http://www.tartaglialab.com/cs_multi/cc_runs/622/; Figure S3), which strongly tightens the link between misfolding and neurodegeneration. In line with this findings, very recent reports showed that increase in protein insolubility is associated with massive accumulation of natively unfolded proteins^28^.

## Conclusions

It has been shown that structurally disordered proteins are tightly regulated by the cell^29^,^30^ and their uncontrolled over-expression triggers pathological conditions such as for instance cardiovascular diseases and diabetes^31^. In this study, we reported the finding that genes up-regulated in CNS diseases are more enriched in disordered protein products than cancer genes, which has important implications for the etiopathogenesis of neurodegenerative diseases. As a matter of fact, changes in the abundance of unfolded proteins induce re-wiring of protein networks and promote formation of aberrant interactions^32^ leading to association with amyloid deposits^26^. As genes up-regulated in prostate, colorectal and lung cancer code for proteins that are less disordered than those up-regulated in CNS diseases and more unfolded than those down-regulated in CNS diseases, we cannot exclude the possibility that structural disorder might play a role in cancer, although to a lesser extent. Indeed, unregulated promiscuity of unfolded proteins can trigger fatal events leading to cell death signalling^29^. For instance, in the case of the Bcl-2 family of apoptosis regulators, aberrant expression of intrinsically disordered proteins can determine different cell fate decisions through alteration of interaction networks^33^ (we note that Bcl-2 is up-regulated in CNS disorders and down-regulated in cancer^2^).

Our results do not indicate that aggregation is uniquely linked to neurodegeneration. Indeed, although amyloid fibrils sequester natively unfolded proteins^26^, which are particularly abundant in brain regions^34^,^35^, some cancer types are associated with protein aggregation^36^ and protein deposits influence cell survival in the context of several tumors, especially those that are metastatic. For example, co-aggregation of toxic amyloid-β peptide (Aβ) and TGF-β-induced antiapoptotic factor (TIAF1) is a hallmark of metastatic cancer cell mass^37^,^38^. Expression levels of TIAF1 vary throughout the metastatic spread, being up-regulated in developing tumors and down-regulated in established metastatic cancer cells^37^. In a number of cases, aggregation of specific genes is associated with both CNS diseases and cancer types. For instance, aggregation of superoxide dismutase SOD1 causes cellular death in amyotrophic lateral sclerosis^39^. Yet, SOD1 has also a role in breast cancer and an ability to augment estrogen-responsive gene expression^40^. Similarly, DNA-binding domain of p53 is conformationally unstable and the majority of disease mutants are known to increase structural disorder^41^. Upon aggregation, mutant p53 not only induces misfolding and co-aggregation of wild-type p53, but also of its paralogues p63 and p73 into cellular inclusions, causing inefficient transcription of target genes, which, in turn, is crucial for cell growth control and apoptosis^42^.

In conclusion, our analysis is one of the first attempts to illustrate how an epidemiological observation on inverse comorbities^2^ can be rationalized in terms of physico-chemical features of proteins encoded by deregulated genes. We cannot exclude that additional factors, including age of disease onset and drug treatment, could influence the expression patterns associated with disease. As a matter of fact, drugs used in the treatment of neurodegenerative diseases, such as for instance thioridazine^43^, have been shown to display anti-tumor effects while anti-tumor drugs, such as cyclin-dependent kinase inhibitors^44^ and mithramycin^45^ are neuro-protective. Yet, these findings reinforce the existence of a link between cancer and CNS diseases and indicate that future studies will have to focus on specific molecular pathways^46^.

## Materials and Methods

Gene sets were taken from the paper by Ibáñez *et al.*^2^: Alzheimer’s disease (AD); Parkinson’s disease (PD); Schizophrenia (SCZ); Colorectal cancer (CRC); Lung cancer (LC); Prostate cancer (PC). Results can be accessed at http://www.tartaglialab.com/cs_multi/confirm/524/36563b35ee/. Examples of our calculations are at http://www.tartaglialab.com/cs_multi/confirm/240/6be82069c3/. Comparison with random sets can be found at http://www.tartaglialab.com/cs_multi/confirm/576/ef217f98eb/ (CNS diseases) and http://www.tartaglialab.com/cs_multi/confirm/602/cfc3e02cdc/ (cancers). Classification of disordered proteins interacting with amyloid fibrils is available at http://www.tartaglialab.com/cs_multi/cc_runs/622/.

### cleverMachine

The cleverMachine (CM) algorithm analyses physico-chemical properties of two protein datasets^10^. The tool creates profiles, or *physico-chemical signatures*, for each protein, utilizing a large set of features - both experimentally and statistically derived from other tools. In our analysis we used a number of physico-chemical properties (hydrophobicity, alpha-helix, beta-sheet, disorder, burial, aggregation, membrane and nucleic acid-binding propensities) and 10 propensity predictors per feature. Only differentially enriched properties were used in the calculations. Further information can be found at http://s.tartaglialab.com/page/clever_suite.

### multiCleverMachine analysis

**Figure 1:**
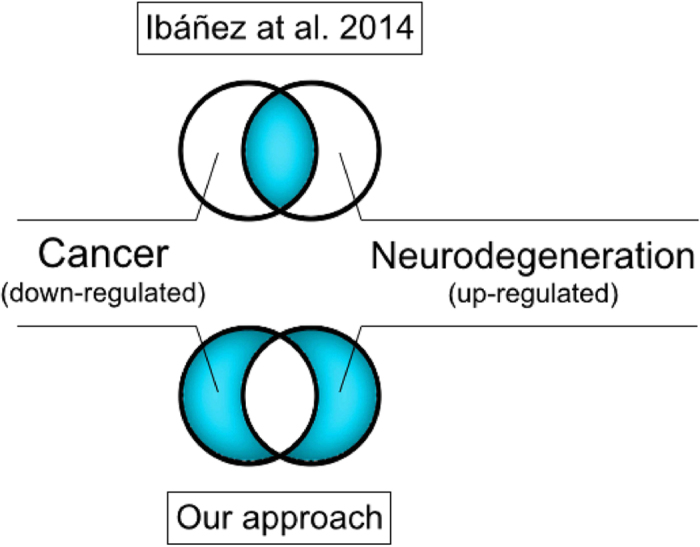
Gene sets analysis. Previous analysis carried out by Ibáñez *et al.*^2^ focused on transcripts that are up-regulated in central nervous system (CNS) and down-regulated in cancer or vice versa (i.e., intersection between gene sets). Our study deals instead with sets of genes that are either up-regulated or down-regulated in cancer and CNS diseases (i.e., symmetric difference between gene sets).

**Figure 2:**
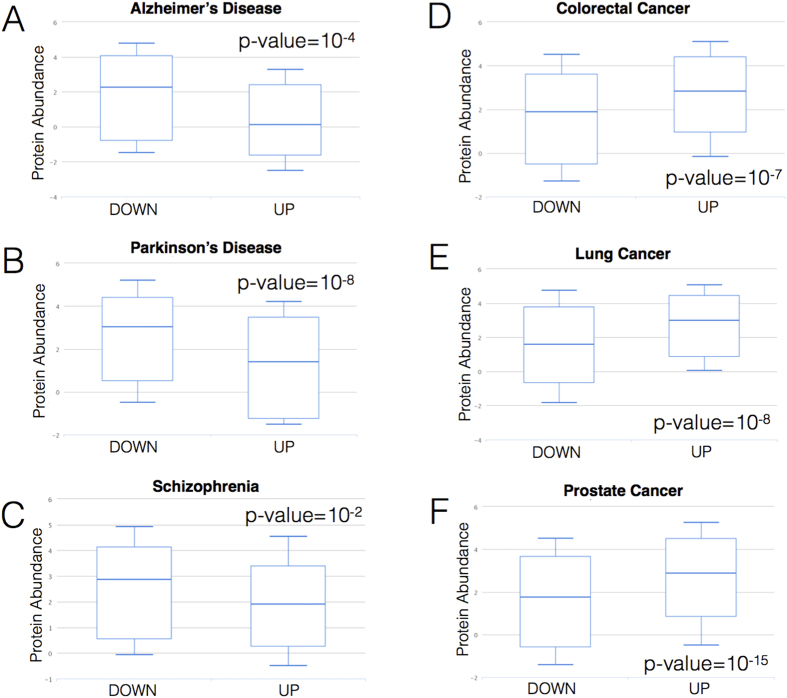
Expression of CNS and cancer genes at physiological conditions. Genes up-regulated (UP) in (**A**) Alzheimer’s, (**B**) Parkinson’s diseases and (**C**) Schizophrenia encode proteins that are poorly abundant under normal conditions^11^,^12^, while down-regulated genes (DOWN) show the opposite trend. Genes up-regulated (UP) in (**D**) Colorectal, (**E**) Lung and (**F**) Prostate cancer encode proteins that are highly abundant in normal conditions, while down-regulated genes (DOWN) show the opposite trend. As physiological concentrations of proteins are linked to their physico-chemical properties^9^,^16^,^19^, our findings reveal information on intrinsic features of disease-associated genes. The p-values are calculated with Kolmogorov-Smirnov test.

The multiCleverMachine (*mult*iCM) extends the concept of binary comparisons used in CM by introducing more set groups. After submission of one or more inputs for signal and one or more inputs as negative group, the multiCM creates a CM run for each possible combination of elements from the signal and negative sets. The result is presented in an easy-to-read format, allowing at a glance interpretation of the CM submissions (Fig. 1). Each of the individual CM runs is linked on the multiCM page, allowing further in-depth analysis. The *multi*CM provides visualisation of enrichment strengths per group, enabling to see easily for which groups the various properties like disorder, alpha-helical propensity, etc. are enriched. Details about this new method are available at http://www.tartaglialab.com/cs_multi/submission.

### DisEMBL analysis

In order to validate our CM analysis, we used DisEMBL^20^ (http://dis.embl.de). As DisEMBL provides disorder profiles for each of the properties, the analysis was carried out as follows. For each of the profiles, we calculated proportion of the sequence that was above the significance threshold defined by the authors, which yielded strength score for each individual entry. The scores were then averaged to compare individual sets. To visualize strength comparisons, we use the same set of colors as described in Fig. 1 (see *multiCleverMachine analysis*): if the set on the left (cancer) has enrichment, the color is green and red otherwise. Our results are available at http://www.tartaglialab.com/static/2014/disembl_analysis.html.

### Age of onset analysis

**Figure 3:**
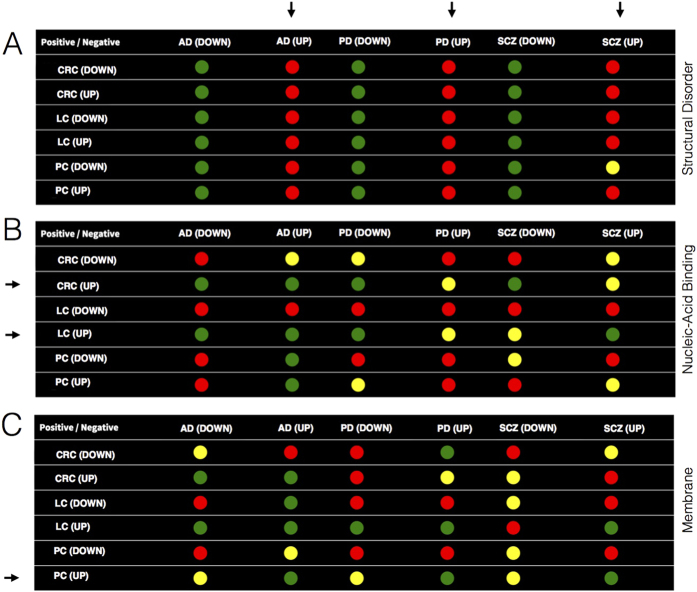
Physico-chemical properties of proteins involved in cancers and CNS diseases. (**A**) Up-regulation of structurally disordered proteins discriminates between cancer types and central nervous system (CNS) diseases. As indicated by horizontal arrows, proteins up-regulated in CNS are enriched in structural disorder (red dots; down-regulation is associated with the opposite trend); (**B**) Nucleic-acid binding propensity differentiates CNS diseases from and proteins up-regulated in colorectal and lung cancer. Proteins up-regulated in colorectal and lung cancer (vertical arrows; green dots) have increased nucleic acid propensity (down-regulation is associated with decrease). (**C**) Membrane propensity differentiates between CNS diseases and proteins up-regulated in prostate cancer. Genes up-regulated in prostate cancer show increased membrane propensity (vertical arrow; green dots; down-regulation is associated with opposite trend). Red: a particular CNS disease is enriched with respect to a cancer type in structural disorder (**A**), nucleic-acid binding propensity (**B**) or membrane propensity (**C**); Green: a cancer type is enriched with respect to a particular CNS disease in structural disorder (**A**), nucleic-acid binding propensity (**B**) or membrane propensity (**C**); Yellow: non significant enrichment; Each enrichment is associated with a p-value < 10^−5^ calculated with Fisher’s exact test; AD: Alzheimer’s disease; PD: Parkinson’s disease; SCZ: Schizophrenia; CRC: Colorectal cancer; LC: Lung cancer; PC: Prostate cancer; UP/DOWN: over/under-expression with respect to healthy control samples.

**Figure 4:**
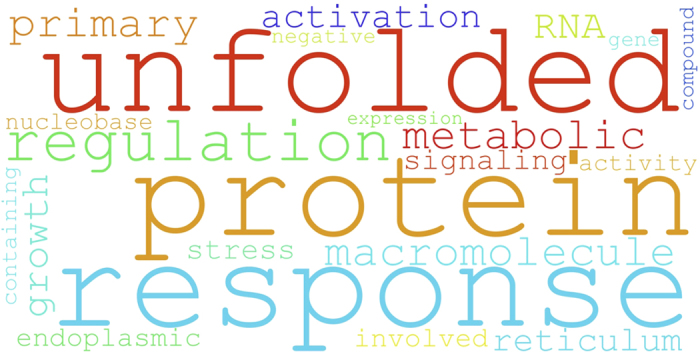
Protein disorder is linked to neurodegeneration. Intrinsically disordered proteins are associated with Gene Ontology (GO) labels that are significantly enriched (p-value < 10^−4^) in terms such as “unfolded protein response” (the example shown refers to Parkinson’s disease genes).

**Figure 5:**
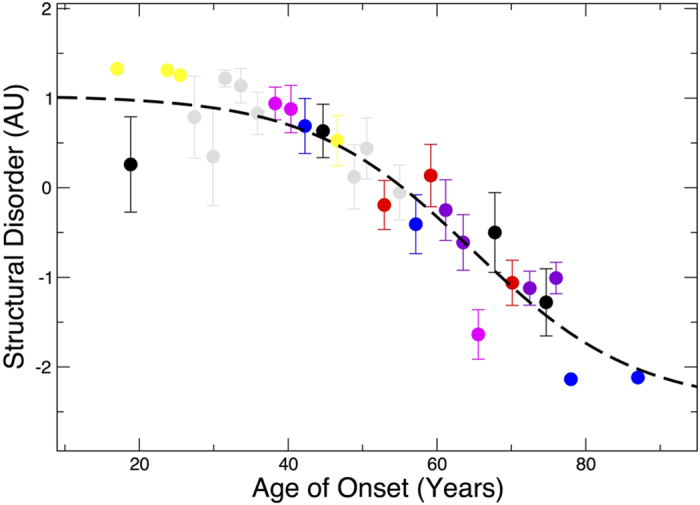
Structural disorder is associated with onset of neurodegenerative diseases. In Frontotemporal Dementia, Alzheimer’s and Parkinson’s diseases, structural disorder is significantly anti-correlated with age of onset (correlation = −0.90; p-value < 10^−3^). A total of 428 mutations and their relative ages of onset grouped with a 2.5 years window have been used for the analysis. Representative genes have been selected to illustrate individual trends (other genes are shown in black): APP, CHMP2B (red), FUS, GRN, LRRK2 (blue), MAPT, PARK2 (yellow), PARK7, PINK1, PSEN1 (gray), PSEN2 (purple), SNCA, TARDBP (pink) and VCP.

We downloaded all single-point amino acid mutations and associated ages of onset from http://www.molgen.ua.ac.be/ADMutations/ and http://www.molgen.vib-ua.be/PDMutDB/. Structural disorder was measured using the B-value propensity scale (linearly normalized between 0 and 1)^24^. For each protein in the dataset, we averaged the disorder propensity over the sequence, as described in our previous publication (values > 0.2)^10^. The relationship between age of onset (AGE) and structural disorder (SD) was assessed with the sigmoidal function 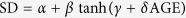 using Z-normalized values for SD (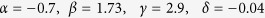; correlation = −0.90; Fig. 3). Using linear regression, 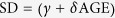, the correlation between SD and AGE is −0.87.

## Additional Information

**How to cite this article**: Klus, P. *et al.* Neurodegeneration and Cancer: Where the Disorder Prevails. *Sci. Rep.* **5**, 15390; doi: 10.1038/srep15390 (2015).

## Supplementary Material

Supplementary Information

## References

[b1] Silva J. L., Gallo C. V. D. M., Costa D. C. F. & Rangel L. P. Prion-like aggregation of mutant p53 in cancer. Trends in Biochemical Sciences 39, 260–267 (2014). 24775734 10.1016/j.tibs.2014.04.001

[b2] Ibáñez K., Boullosa C., Tabarés-Seisdedos R., Baudot A. & Valencia A. Molecular Evidence for the Inverse Comorbidity between Central Nervous System Disorders and Cancers Detected by Transcriptomic Meta-analyses. PLoS Genet 10, e1004173 (2014). 24586201 10.1371/journal.pgen.1004173 PMC3930576

[b3] Garber K. Parkinson’s Disease and Cancer: The Unexplored Connection. JNCI J Natl Cancer Inst 102, 371–374 (2010). 20215596 10.1093/jnci/djq081

[b4] Roe C. M. *et al.* Cancer linked to Alzheimer disease but not vascular dementia. Neurology 74, 106–112 (2010). 20032288 10.1212/WNL.0b013e3181c91873 PMC2809029

[b5] Wilhelm M. *et al.* Mass-spectrometry-based draft of the human proteome. Nature 509, 582–587 (2014). 24870543 10.1038/nature13319

[b6] Cirillo D. *et al.* Constitutive patterns of gene expression regulated by RNA-binding proteins. Genome Biol. 15, R13 (2014). 24401680 10.1186/gb-2014-15-1-r13 PMC4054784

[b7] Tartaglia G. G., Dobson C. M., Hartl F. U. & Vendruscolo M. Physicochemical determinants of chaperone requirements. J. Mol. Biol 400, 579–588 (2010). 20416322 10.1016/j.jmb.2010.03.066

[b8] Tartaglia G. G. & Vendruscolo M. Correlation between mRNA expression levels and protein aggregation propensities in subcellular localisations. Mol. BioSyst. 5, 1873–1876 (2009). 19763336 10.1039/b913099n

[b9] Tartaglia G. G., Pechmann S., Dobson C. M. & Vendruscolo M. Life on the edge: a link between gene expression levels and aggregation rates of human proteins. Trends Biochem Sci 32, 204–6 (2007). 17419062 10.1016/j.tibs.2007.03.005

[b10] Klus P. *et al.* The cleverSuite approach for protein characterization: predictions of structural properties, solubility, chaperone requirements and RNA-binding abilities. Bioinformatics 30, 1601–1608 (2014). 24493033 10.1093/bioinformatics/btu074 PMC4029037

[b11] Desiere F. *et al.* The PeptideAtlas project. Nucl. Acids Res. 34, D655–D658 (2006). 16381952 10.1093/nar/gkj040 PMC1347403

[b12] Wang M. *et al.* PaxDb, a database of protein abundance averages across all three domains of life. Mol Cell Proteomics (2012). 10.1074/mcp.O111.014704. PMC3412977 22535208

[b13] Tartaglia G. G. & Caflisch A. Computational analysis of the S. cerevisiae proteome reveals the function and cellular localization of the least and most amyloidogenic proteins. Proteins 68, 273–8 (2007). 17407164 10.1002/prot.21427

[b14] Baldwin A. J. *et al.* Metastability of native proteins and the phenomenon of amyloid formation. J. Am. Chem. Soc. 133, 14160–14163 (2011). 21650202 10.1021/ja2017703

[b15] Bolognesi B. & Tartaglia G. G. Physicochemical principles of protein aggregation. Prog Mol Biol Transl Sci 117, 53–72 (2013). 23663965 10.1016/B978-0-12-386931-9.00003-9

[b16] Edwards Y. J. K., Lobley A. E., Pentony M. M. & Jones D. T. Insights into the regulation of intrinsically disordered proteins in the human proteome by analyzing sequence and gene expression data. Genome Biol. 10, R50 (2009). 19432952 10.1186/gb-2009-10-5-r50 PMC2718516

[b17] Vavouri T., Semple J. I., Garcia-Verdugo R. & Lehner B. Intrinsic Protein Disorder and Interaction Promiscuity Are Widely Associated with Dosage Sensitivity. Cell 138, 198–208 (2009). 19596244 10.1016/j.cell.2009.04.029

[b18] Mittal N., Roy N., Babu M. M. & Janga S. C. Dissecting the expression dynamics of RNA-binding proteins in posttranscriptional regulatory networks. Proc. Natl. Acad. Sci. USA 106, 20300–20305 (2009). 19918083 10.1073/pnas.0906940106 PMC2777960

[b19] Kechavarzi B. & Janga S. C. Dissecting the expression landscape of RNA-binding proteins in human cancers. Genome Biol. 15, R14 (2014). 24410894 10.1186/gb-2014-15-1-r14 PMC4053825

[b20] Linding R. *et al.* Protein disorder prediction: implications for structural proteomics. Structure 11, 1453–1459 (2003). 14604535 10.1016/j.str.2003.10.002

[b21] Marsh J. A. Buried and Accessible Surface Area Control Intrinsic Protein Flexibility. Journal of Molecular Biology 425, 3250–3263 (2013). 23811058 10.1016/j.jmb.2013.06.019

[b22] Liu J. *et al.* Intrinsic disorder in transcription factors. Biochemistry 45, 6873–6888 (2006). 16734424 10.1021/bi0602718 PMC2538555

[b23] Bera T. K. *et al.* NGEP, a gene encoding a membrane protein detected only in prostate cancer and normal prostate. PNAS 101, 3059–3064 (2004). 14981236 10.1073/pnas.0308746101 PMC365744

[b24] Campen A. *et al.* TOP-IDP-scale: a new amino acid scale measuring propensity for intrinsic disorder. Protein Pept Lett 15, 956–63 (2008). 18991772 10.2174/092986608785849164 PMC2676888

[b25] Fink A. L. The Aggregation and Fibrillation of α-Synuclein. Acc. Chem. Res. 39, 628–634 (2006). 16981679 10.1021/ar050073t

[b26] Olzscha H. *et al.* Amyloid-like Aggregates Sequester Numerous Metastable Proteins with Essential Cellular Functions. Cell 144, 67–78 (2011). 21215370 10.1016/j.cell.2010.11.050

[b27] Soares D. C., Carlyle B. C., Bradshaw N. J. & Porteous D. J. DISC1: Structure, Function, and Therapeutic Potential for Major Mental Illness. ACS Chem Neurosci 2, 609–632 (2011). 22116789 10.1021/cn200062k PMC3222219

[b28] Walther D. M. *et al.* Widespread Proteome Remodeling and Aggregation in Aging C. elegans. Cell 161, 919–932 (2015). 25957690 10.1016/j.cell.2015.03.032 PMC4643853

[b29] Gsponer J., Futschik M. E., Teichmann S. A. & Babu M. M. Tight regulation of unstructured proteins: from transcript synthesis to protein degradation. Science 322, 1365–1368 (2008). 19039133 10.1126/science.1163581 PMC2803065

[b30] Babu M. M., van der Lee R., de Groot N. S. & Gsponer J. Intrinsically disordered proteins: regulation and disease. Curr. Opin. Struct. Biol. 21, 432–440 (2011). 21514144 10.1016/j.sbi.2011.03.011

[b31] Uversky V. N. Wrecked regulation of intrinsically disordered proteins in diseases: pathogenicity of deregulated regulators. Front. Mol. Biosci 1, 6 (2014). 25988147 10.3389/fmolb.2014.00006 PMC4428494

[b32] Turoverov K. K., Kuznetsova I. M. & Uversky V. N. The protein kingdom extended: ordered and intrinsically disordered proteins, their folding, supramolecular complex formation, and aggregation. Prog. Biophys. Mol. Biol. 102, 73–84 (2010). 20097220 10.1016/j.pbiomolbio.2010.01.003 PMC2916636

[b33] Rautureau G. J. P., Day C. L. & Hinds M. G. Intrinsically Disordered Proteins in Bcl-2 Regulated Apoptosis. Int J Mol Sci 11, 1808–1824 (2010). 20480043 10.3390/ijms11041808 PMC2871139

[b34] Weatheritt R. J., Gibson T. J. & Babu M. M. Asymmetric mRNA localization contributes to fidelity and sensitivity of spatially localized systems. Nat Struct Mol Biol 21, 833–839 (2014). 25150862 10.1038/nsmb.2876 PMC4167633

[b35] Levine Z. A., Larini L., LaPointe N. E., Feinstein S. C. & Shea J.-E. Regulation and aggregation of intrinsically disordered peptides. PNAS 112, 2758–2763 (2015). 25691742 10.1073/pnas.1418155112 PMC4352815

[b36] De Oliveira G. A. P., Rangel L. P., Costa D. C. & Silva J. L. Misfolding, aggregation, and disordered segments in c-Abl and p53 in human cancer. Front. Oncol. 97 (2015). 10.3389/fonc.2015.00097 25973395 PMC4413674

[b37] Chang J.-Y. *et al.* TIAF1 self-aggregation in peritumor capsule formation, spontaneous activation of SMAD-responsive promoter in p53-deficient environment, and cell death. Cell Death Dis 3, e302 (2012). 22534828 10.1038/cddis.2012.36 PMC3358014

[b38] Hong Q. *et al.* Self-aggregating TIAF1 in lung cancer progression. Translational Respiratory Medicine 1, 5 (2013). 10.1186/2213-0802-1-5 PMC6733429 27234387

[b39] Cleveland D. W. & Rothstein J. D. From Charcot to Lou Gehrig: deciphering selective motor neuron death in ALS. Nat. Rev. Neurosci. 2, 806–819 (2001). 11715057 10.1038/35097565

[b40] Stathopulos P. B. *et al.* Cu/Zn superoxide dismutase mutants associated with amyotrophic lateral sclerosis show enhanced formation of aggregates *in vitro*. Proc. Natl. Acad. Sci. USA 100, 7021–7026 (2003). 12773627 10.1073/pnas.1237797100 PMC165823

[b41] Ang H. C., Joerger A. C., Mayer S. & Fersht A. R. Effects of common cancer mutations on stability and DNA binding of full-length p53 compared with isolated core domains. J. Biol. Chem. 281, 21934–21941 (2006). 16754663 10.1074/jbc.M604209200

[b42] Xu J. *et al.* Gain of function of mutant p53 by coaggregation with multiple tumor suppressors. Nat Chem Biol 7, 285–295 (2011). 21445056 10.1038/nchembio.546

[b43] Sachlos E. *et al.* Identification of drugs including a dopamine receptor antagonist that selectively target cancer stem cells. Cell 149, 1284–1297 (2012). 22632761 10.1016/j.cell.2012.03.049

[b44] Park D. S. *et al.* Cyclin-dependent kinases participate in death of neurons evoked by DNA-damaging agents. J. Cell Biol. 143, 457–467 (1998). 9786955 10.1083/jcb.143.2.457 PMC2132832

[b45] Sleiman S. F. *et al.* Mithramycin Is a Gene-Selective Sp1 Inhibitor That Identifies a Biological Intersection between Cancer and Neurodegeneration. J Neurosci 31, 6858–6870 (2011). 21543616 10.1523/JNEUROSCI.0710-11.2011 PMC3717375

[b46] Plun-Favreau H., Lewis P. A., Hardy J., Martins L. M. & Wood N. W. Cancer and Neurodegeneration: Between the Devil and the Deep Blue Sea. PLoS Genet 6, (2010). 10.1371/journal.pgen.1001257 PMC3009676 21203498

