# Supplementary material for: Neurodegeneration and Cancer: Where the Disorder Prevails
Source: Sci Rep. 2015 Oct 23;5:15390. doi: 10.1038/srep15390 (PMC4615981; doi:10.1038/srep15390)
Supplement: Supplementary Information [file srep15390-s1.pdf]

Supplementary Information of

Neurodegeneration and Cancer: Where the Disorder Prevails

Petr Klus[1,2], Davide Cirillo[1,2], Teresa Botta Orfila[1,2] and Gian Gaetano Tartaglia[1,2,3,*]

[1] Centre for Genomic Regulation (CRG), Dr Aiguader 88, 08003 Barcelona, Spain.

[2] Universitat Pompeu Fabra (UPF), 08003 Barcelona, Spain

[3] Institució Catalana de Recerca i Estudis Avançats (ICREA), 23 Passeig Lluís Companys, 08010 Barcelona, Spain

* Corresponding Author. Tel: +34 933160116; Fax: +34 933969983; Email: gian.tartaglia@crg.eu

CDS diseases (abundance and structural disorder)
AD_DOWN (positive) vs AD_UP (negative)
http://www.tartaglialab.com/boxplotter/view/629/a6f02b1232/

PD_DOWN (pos) vs PD_UP (neg)
http://www.tartaglialab.com/boxplotter/view/631/c68cb71821/

SCZ_DOWN (pos) vs. SCZ_UP (neg)
http://www.tartaglialab.com/boxplotter/view/636/23064d250f/

Cancers (abundance and nucleic-acid binding propensity)
CRC_DOWN (pos) vs. CRC_UP (neg)
http://www.tartaglialab.com/boxplotter/view/643/8e2fd086a3/

LC_DOWN (pos) vs. LC_UP (neg)
http://www.tartaglialab.com/boxplotter/view/644/c70a66dc46/

PC_DOWN (pos) vs. PC_UP (neg)
http://www.tartaglialab.com/boxplotter/view/639/0f2a9ac422/

Table S1. *Protein abundance analysis.* We provide links to the statistical analysis of genes sets performed with *boxplotter* (http://www.tartaglialab.com/boxplotter/submit)

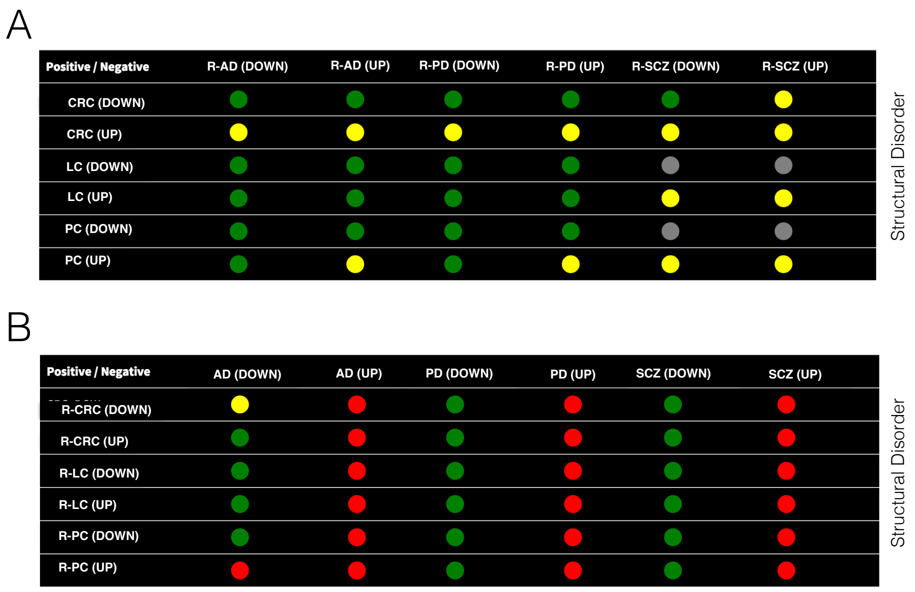

Figure S1. *Enrichments in structural disorder propensities.* We used random sets of genes to measure the signal strength of structural disordered propensities for A) Cancers B) CNS diseases.

FileA/FileB	AD (DOWN)	AD (UP)
CRC (DOWN)	N 0.90	P 0.60
CRC (UP)		P 0.76
LC (DOWN)	N 0.79	P 0.71
LC (UP)	N 0.77	P 0.71
PC (DOWN)	N 0.80	P 0.56
PC (UP)	N 0.83	P 0.71

Figure S2. *Classification of proteins binding to amyloid fibrils.* Proteins interacting with amyloid fibrils are physico-chemically similar to those overexpressed in Alzheimer's disease, in agreement with experimental findings.
